# On the genus *Sericopimpla* Kriechbaumer, 1895 (Ichneumonidae, Pimplinae) in the Afrotropics, with the description of a new species

**DOI:** 10.3897/zookeys.1268.181918

**Published:** 2026-02-10

**Authors:** Emil M. Österman, Tapani Hopkins, Ilari E. Sääksjärvi, Kari M. Kaunisto, Simon van Noort

**Affiliations:** 1 Biodiversity Unit, Zoological Museum, University of Turku, 20014 Turku, Finland University of Cape Town Rondebosch South Africa; 2 Research and Exhibitions Department, South African Museum, Iziko Museums of South Africa, PO Box 61, Cape Town, 8000, South Africa South African Museum, Iziko Museums of South Africa Cape Town South Africa; 3 Department of Biological Sciences, University of Cape Town, Private Bag Rondebosch, Rondebosch, Cape Town 7701, South Africa University of Turku Turku Finland

**Keywords:** Africa, biodiversity, Darwin wasps, Kibale National Park, Malaise trap, morphological variation, taxonomy, tropical forest

## Abstract

*Sericopimpla* Kriechbaumer, 1895 (Hymenoptera, Ichneumonidae, Pimplinae) is a moderately large Darwin wasp genus distributed mostly in the Paleotropics and Australasian region. Here, we describe a new Afrotropical species of the genus, *S. kibalensis***sp. nov**., and provide notes on the intraspecific variation of the other known Afrotropical species: *S. sericata* (Kriechbaumer, 1895). We record the genus for the first time from Uganda, based on specimens collected with Malaise traps in Kibale National Park.

## Introduction

*Sericopimpla* Kriechbaumer, 1895 is a moderately large genus within the tribe Ephialtini of the Pimplinae subfamily of Darwin wasps (Hymenoptera, Ichneumonidae). It has a wide distribution, occurring in the Palaeotropics and Australasian region, as well as a few species in the northeast, temperate Palaearctic region, for example, *S. maculata* Sheng & Sun, 2014 from Liaoning, China (Yu et al. 2016). Most species (12) have been described from the Indo-Malayan region (Snellen van Vollenhoven 1879; Cameron 1902; Morley 1913; Baltazar 1961; Gupta and Tikar 1976; Sathe et al. 1990). Previously, the genus included 21 species (Snellen van Vollenhoven 1879; Cameron 1902; Morley 1913, 1916; Brues 1918; Baltazar 1961; Momoi 1970, 1977; Gupta and Tikar 1976; Gauld 1984; Sathe et al. 1990; Sheng and Sun 2014; Yu et al. 2016), of which only the widely distributed *S. sericata* (Kriechbaumer, 1895) is Afrotropical (Kriechbaumer 1895; Szépligeti 1908; Roman 1910; Morley 1916; Benoit 1953, 1955, 1956; Townes and Townes 1973). The usual hosts of species of *Sericopimpla* are bagworm moths (Lepidoptera, Psychidae) (Townes 1969; Gauld 1984). However, only the biology of one species, *S. sericata*, seems to have been studied in detail. Smithers (1956) noted that *S. sericata* developed as an (apparently solitary) idiobiont ectoparasitoid on *Acanthopsyche junodi* (Heylaerts, 1890) (= *Kotochalia junodi*) larvae. In addition, Gauld (1984) reported that the Oriental *S. albicincta* (Morley, 1913) is gregarious, with 15 individuals observed to emerge from the same host.

*Sericopimpla* can easily be distinguished from other pimpline genera by the combination of the following characteristics: eye strongly indented opposite antennal socket (Fig. 8); malar space nearly closed (Fig. 2); occipital carina complete; fore wing with closed, subtriangular areolet (Fig. 4); hind wing with abscissa of *CU* between *M* and *cu-a* longer than *cu-a* (Fig. 4) (diagnosis modified from Gauld 1984). Of the pimpline genera occurring in the Afrotropical region, *Itoplectis* Förster, 1869 may superficially resemble *Sericopimpla* in also having a strongly indented eye opposite the antennal socket and some species having a relatively short malar space. However, *Itoplectis* differs in other characteristics, for example, in not having a distinctly subtriangular areolet and, as a member of the Pimplini tribe, in having the hind wing with the abscissa of *CU* between *M* and *cu-a* shorter than *cu-a*.

In this article, we describe a new species, *S. kibalensis* sp. nov., and provide notes on the morphological variation of *S. sericata* from Uganda, where the genus is recorded for the first time. This article is part of a series of publications describing new pimpline species collected in an extensive one-year sampling in Kibale (reported by Hopkins et al. 2019a; Österman et al. 2025a).

## Methods

The Ugandan *Sericopimpla* specimens studied here were separated from samples collected with 34 Malaise traps that operated for one year (2014–2015) in Kibale National Park, Uganda. The traps were placed in various habitats that varied across a successional gradient from primary forest to farmland. The sampling effort totaled 382.4 trap months, of which 22.6 trap months were damaged samples or otherwise provided at most a partial catch of Pimplinae. The study area and sampling campaign are described in greater detail by Hopkins et al. (2019a, b).

We photographed the Ugandan specimens at the Zoological Museum, Turku (**ZMUT**) using a Sony Alpha 9 Mark II camera body mounted on a macro rail, which enabled us to control and incrementally move the camera between shots. Photographs were captured using an extension tube, a relay lens, and Mitutoyo Plan Apo objectives with magnifications ranging from 2.5× to 5×. We captured multiple images at successive focal depths and combined them using the software Helicon Focus v. 7.7.5 (Helicon Soft Ltd) to produce composite layer images with extended depth of field. We carried out final image adjustments in Adobe Photoshop CC to ensure accurate representation of the specimen’s morphological features. Observation at ZMUT were made using Olympus SZ61 and SZX16 stereomicroscopes.

We acquired images at the South African Museum, Cape Town (SAMC) using a Leica LAS v. 4.9 imaging system, comprising a Leica® Z16 microscope (using either a 2× or 5× objective) with a Leica DFC450 Camera and 0.63× video objective attached; we achieved diffused lighting using a Leica LED5000 HDI dome; and the imaging process, using an automated Z-stepper, which we managed using the Leica Application Suite v. 4.9 software installed on a desktop computer.

Morphological terminology follows Broad et al. (2018), except for male genitalia that follow Dal Pos et al. (2023). We based our species description largely on the format used by Gauld (1984) to describe species of *Sericopimpla*. To present all available information about the examined specimens of *Sericopimpla sericata*, we photographed their labels and cited the information verbatim with notes added in square brackets.

### Repositories

**SAMC** South African Museum, Iziko Museums of South Africa, Cape Town, South Africa (Simon van Noort);

**ZMUT** Zoological Museum, Biodiversity Unit, University of Turku, Turku, Finland (Ilari Sääksjärvi).

## Results

### Taxonomy

#### Sericopimpla kibalensis

Taxon classificationAnimaliaHymenopteraIchneumonidae

Österman & Sääksjärvi sp. nov.

CBEBA2BC-F16B-53AE-BFE1-9AE4439858E8

https://zoobank.org/19068D98-D853-4040-A64E-6A369678DB75

Figs 1, 2, 3, 4

##### Material examined.

Uganda, Kibale National Park, Kanyawara; Tapani Hopkins leg; ZMUT:

##### Type material.

• ***Holotype***, 1 female. Site K30S, Malaise trap K30ST4; 0.5414°N, 30.3755°E; alt. 1421 m; 15–26 Dec. 2014; http://mus.utu.fi/ZMUT.2363. • ***Paratype***, 1 female. Site K13, Malaise trap K13T3; 0.5927°N, 30.3616°E; alt. 1492 m; 1–15 Jan. 2015; http://mus.utu.fi/ZMUT.3514 • ***Paratype***, 1 female. Site K13, Malaise trap K13T3; 0.5927°N, 30.3616°E; alt. 1492 m; 12–26 Jan. 2015; http://mus.utu.fi/ZMUT.3581 • ***Paratype***, 2 females. Site K15, Malaise trap K15T2; 0.5843°N, 30.3644°E; alt. 1471 m; 12–26 Jan. 2015; http://mus.utu.fi/ZMUT.10774, http://mus.utu.fi/ZMUT.10778 • ***Paratype***, 1 male. Site K31, Malaise trap K31T3; 0.5360°N, 30.3469°E; alt. 1449 m; 30 Jan.–13 Feb. 2015; http://mus.utu.fi/ZMUT.1603. • ***Paratype***, 1 male. Site R03, Malaise trap R03T2; 0.5402°N, 30.3608°E; alt. 1491 m; 29 Dec. 2014–15 Jan. 2015; http://mus.utu.fi/ZMUT.2001 • ***Paratype***, 2 males. Site K31, Malaise trap K31T4; 0.5362°N, 30.3486°E; alt. 1463 m; 30 Jan.–13 Feb. 2015; http://mus.utu.fi/ZMUT.2853, http://mus.utu.fi/ZMUT.2881.

##### Non-type material.

(only diagnostic characters checked) • 23 females. Malaise traps; http://mus.utu.fi/ZMUT.1722, http://mus.utu.fi/ZMUT.2199, http://mus.utu.fi/ZMUT.5401, http://mus.utu.fi/ZMUT.13621, http://mus.utu.fi/ZMUT.17188, http://mus.utu.fi/ZMUT.17585, http://mus.utu.fi/ZMUT.17670, http://mus.utu.fi/ZMUT.18071, http://mus.utu.fi/ZMUT.18270, http://mus.utu.fi/ZMUT.20366, http://mus.utu.fi/ZMUT.20757, http://mus.utu.fi/ZMUT.20866, http://mus.utu.fi/ZMUT.24974, http://mus.utu.fi/ZMUT.26569, http://mus.utu.fi/ZMUT.26858, http://mus.utu.fi/ZMUT.26964, http://mus.utu.fi/ZMUT.28459, http://mus.utu.fi/ZMUT.28484, http://mus.utu.fi/ZMUT.28653, http://mus.utu.fi/ZMUT.28654, http://mus.utu.fi/ZMUT.28918, http://mus.utu.fi/ZMUT.29033, http://mus.utu.fi/ZMUT.29298. • 14 males. Malaise traps; http://mus.utu.fi/ZMUT.16584, http://mus.utu.fi/ZMUT.17162, http://mus.utu.fi/ZMUT.17164, http://mus.utu.fi/ZMUT.17619, http://mus.utu.fi/ZMUT.17620, http://mus.utu.fi/ZMUT.17929, http://mus.utu.fi/ZMUT.17966, http://mus.utu.fi/ZMUT.18335, http://mus.utu.fi/ZMUT.20370, http://mus.utu.fi/ZMUT.25544, http://mus.utu.fi/ZMUT.26957, http://mus.utu.fi/ZMUT.27450, http://mus.utu.fi/ZMUT.27461, http://mus.utu.fi/ZMUT.27465.

##### Diagnosis.

This new species can be distinguished from all other species of the genus by the combination of the following characteristics: scape and pedicel ventrally white; mesoscutum, other than margins, aciculate and with coarse punctures separated by less than their own diameter; propodeum coarsely punctate (except for mostly smooth metapleuron); ovipositor projecting beyond apex of metasoma by about 1.5–1.6× length of hind tibia; hind tibia, other than basal 0.2, and tarsus yellowish; metasoma mostly orange or yellowish with only basal tergites black-marked.

**Figures 1–3.**
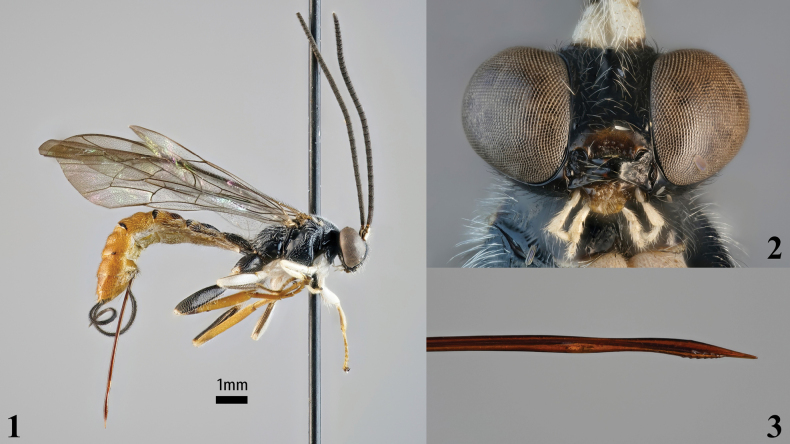
*Sericopimpla kibalensis* sp. nov., holotype female (ZMUT http://mus.utu.fi/ZMUT.2363). **1**. Habitus, lateral view; **2**. Head, anterior view; **3**. Ovipositor apex, lateral view.

**Figure 4.**
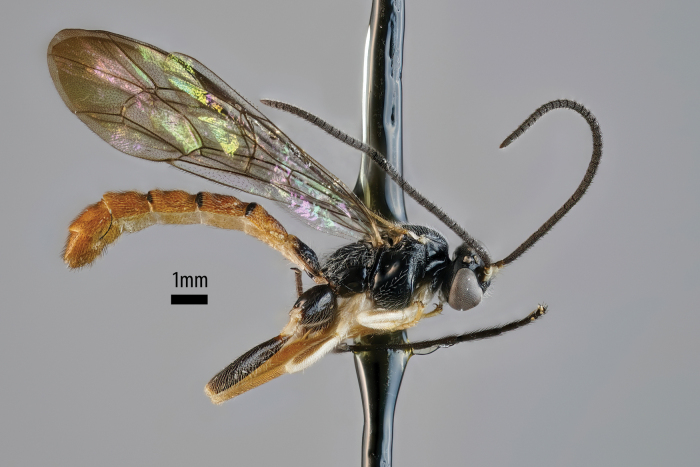
*Sericopimpla kibalensis* sp. nov., paratype male (ZMUT http://mus.utu.fi/ZMUT.1603), habitus in lateral view.

##### Description.

**Female**: forewing length about 8.3 mm. ***Head*** polished. Mandible base nearly aligned with vertical plane; mandible basally very strongly tapered, otherwise weakly tapered, with teeth equal. Lower face (just above clypeus) about 0.9× as wide as medially high, bearing very weak setiferous punctures. Upper face (just below antennal sockets) with weak vertical swelling. Flagellum with 33 flagellomeres, F1 about 1.6–1.7× as long as F2. Head in dorsal view with gena narrowed and almost straight. Lateral ocellus separated from compound eye by about 0.7× its own maximum diameter. ***Mesosoma*** polished. Pronotum mostly bare, with setiferous punctures along dorsal margin; epomia very short. Mesoscutum aciculate and with coarse setiferous punctures, with distance between punctures smaller than their diameter, margins smooth with small setiferous punctures; notaulus rather weakly impressed. Scutellum with long setae arising from coarse punctures. Mesopleuron covered with setiferous punctures, except for bare antero-ventral margin and oblique posterior area, with coarse punctures on upper epicnemium and near subalar prominence; epicnemial carina reaching the level of the middle of the posterior margin of the pronotum. Metapleuron bare except for weak setiferous punctures on dorsal margin (sparse) and posterior part, with lower anterior corner flanged and bent outward; submetapleural carina complete. Propodeum in profile quite strongly and evenly arched, covered with coarse punctures with long setae, with distance between punctures smaller than their diameter; propodeum with complete pleural carina and posteriorly with short lateral longitudinal carina. Fore wing vein 1*cu-a* opposite base of *M&RS*. Hind wing with abscissa of *CU* between *M* and *cu-a* straight, about 1.2× as long as *cu-a*. ***Metasoma*** polished. Tergite 1 in profile rather low, declivous most basally, otherwise flat, about 1.2× as long as apically broad, latero-median carinae absent. Tergite 2 about as long as apically broad. Tergites 1–5 covered with coarse setiferous punctures except for at basal and apical margins. Tergites 6+ with weak setiferous punctures. Tergites 3–5 with distinct latero-median tubercles. Ovipositor straight, projecting beyond apex of metasoma by about 1.5–1.6× length of hind tibia; ventral valve strongly dorso-ventrally constricted basal of teeth, dorsal valve distinctly dorso-ventrally constricted about twice the distance of nodus to apex basal of nodus; apex of lower valve bearing about 6 oblique teeth.

***Colour***: mostly white-haired, hairs especially conspicuous in head and mesosoma (especially propodeum). Head black with white palps and ventral scape and pedicel, clypeus brownish except for dorsal margin. Mesosoma black with white hind corner of pronotum, tegula, and centrally interrupted band along posterior margin of scutellum and postscutellum; fore leg white with blackish dorsal femur and ventral tibia and tarsus; mid leg white with blackish dorsal femur, tibia and ventral tarsus; hind leg yellowish with black coxa, femur and antero-lateral patch on basal 0.2 of tibia, white trochanter, ventral trochantellus and antero-lateral base of femur; wings mostly uncoloured with apical cells in forewing infumate, pterostigma black. Metasoma yellowish with black tergite 1, median raised area and lateral band of tergites 2–3 (weaker on tergite 3), band along posterior margin of tergites 2–4, which is increasingly centrally interrupted in more apical tergites, and ovipositor sheath, white tergite 2 except for black areas, anterior tergite 3 and membranous areas of sternites.

**Male**. Similar to female in structure, but slightly smaller (forewing length 5.2–7.0 mm); flagellum with 27–29 flagellomeres; gonostyle with long hairs. Colour similar to female, but tergites 2–3 more unicolor (orange, with dark posterior marks), without whitish areas and with at most two small brownish marks centrally.

##### Etymology.

This species is named after its type locality: Kibale National Park, Uganda, Africa.

##### Distribution.

Uganda (only known from Kibale National Park).

##### Biological notes.

The host(s) are unknown but could be bagworm moths (see Discussion). The species may mainly live in forest, since it was caught in a variety of forest habitats in Kibale National Park, but not in farmland outside the forest. Its phenology in Malaise traps in Kibale National Park followed what seems to be a common pattern for idiobiont ectoparasitoids, peaking in the two dry seasons. Males were almost only caught toward the end of the first (December to February) dry season.

##### Variation.

Forewing length 7.3–9.2 mm and 30–33 flagellomeres in females. The colour of the hind coxa and femur varies between black and brown, and the black areas on tergites 3+ vary in strength (a few specimens have only the apical margin black on tergite 3). The colour of male tergite 2 varies from largely orange with two posterior brownish marks (most males) to largely orange with two central brownish marks and two posterior brownish marks.

##### Remarks.

This new species is morphologically similar to *S. sericata*, its only known Afrotropical congener, but it can be separated from it by any of the following characteristics: scape and pedicel ventrally white (black in *S. sericata*); hind tarsus and tibia mainly yellowish (black in *S. sericata*); female metasomal tergites 3+ mainly yellowish (mainly reddish in *S. sericata* females).

#### Sericopimpla sericata

Taxon classificationAnimaliaHymenopteraIchneumonidae

(Kriechbaumer, 1895)

EF23DAC5-C248-553A-A65E-797B60D20048

Figs 5, 6, 7, 8, 9, 10, 11, 12, 13, 14

Pimpla (Sericopimpla) sericata Kriechbaumer, 1895: 135. Holotype female, Mozambique (lost)
Pimpla areolaris Szépligeti, 1908: 81. Lectotype female (designated by Townes and Townes 1973), Tanzania (NHRS)
Exeristes areolaris occidentalis Roman, 1910: 161. Lectotype female (designated by Townes and Townes 1973), Democratic Rep. of Congo (NHRS)
Philopsyche abdominalis Morley, 1916: 388. Lectotype female (designated by Townes and Townes 1973), South Africa (SAMC)

##### Diagnosis.

This species can be distinguished from all other species of the genus by the combination of the following characteristics: scape and pedicel ventrally black; mesoscutum, other than margins, aciculate and with coarse punctures separated by less than their own diameter; propodeum coarsely punctate (except for mostly smooth metapleuron); ovipositor projecting beyond apex of metasoma by about 1.6–1.7× length of hind tibia; hind leg blackish; metasoma reddish in female and orange in male, occasionally with basal tergites black-marked.

**Figures 5–8.**
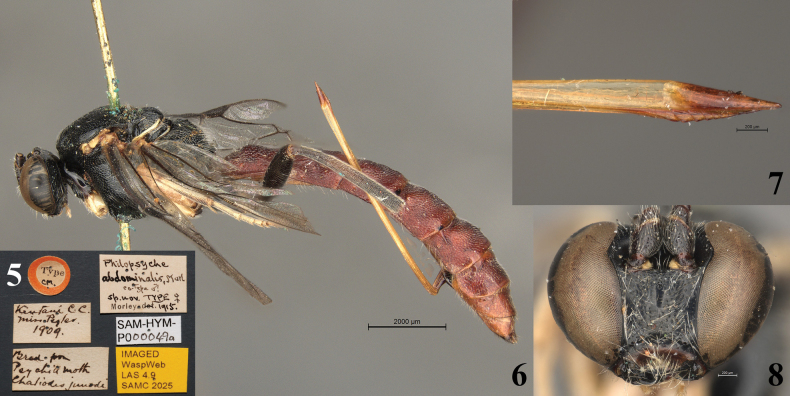
*Philopsyche abdominalis* (= *Sericopimpla sericata*), lectotype female (SAMC). **5**. Data labels; **6**. Habitus, lateral view; **7**. Ovipositor apex, lateral view; **8**. Head, anterior view.

**Figures 9, 10.**
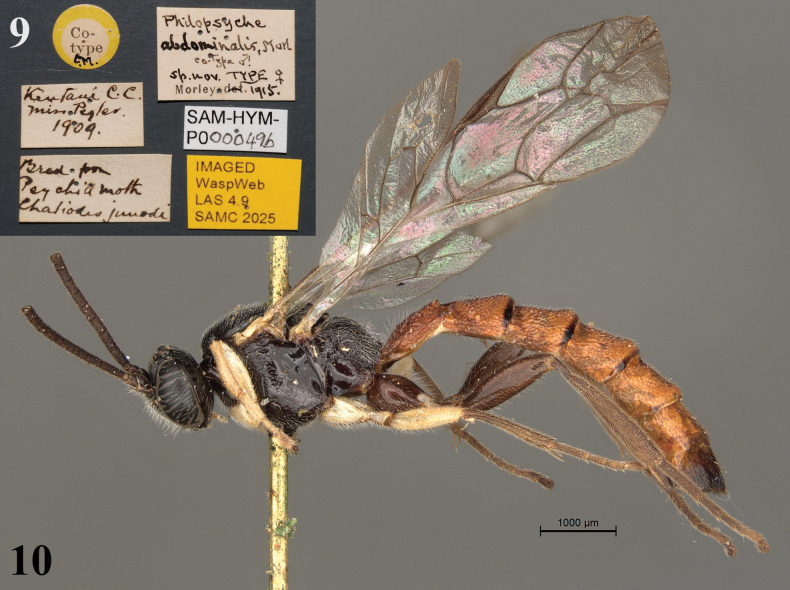
*Philopsyche abdominalis* (= *Sericopimpla sericata*), paralectotype male (SAMC). **9**. Data labels; **10**. Habitus, lateral view.

##### Material examined.

**Type material**: • ***lectotype****Philopsyche abdominalis* Morley, 1916 (designated by Townes and Townes 1973), female, Type CM [in small white circle bordered by red], [South Africa, Eastern Cape Province], Kentani, C.C. [= Cape Colony], [−32.505, 28.314], Miss Pegler 1909, Bred from Psychid moth, *Chaliodes junodi* [= *Kotochalia junodi*] (SAMC). • *Philopsyche abdominalis*, Morl. co-type male, sp. nov., TYPE female, Morley 1915, IMAGED WaspWeb LAS 4.9 SAMC 2025, SAM-HYM-P000049a (SAMC); paralectotype, 1 male, ditto except for co-type CM [in small white circle bordered by yellow], SAM-HYM-P000049b (SAMC).

##### Non-type material.

• 1 female, [South Africa, Kwazulu-Natal Province], Clan Syndicate, Natal, VI-25-14 [= 25 June 1914], C.B. Hardenberg, W.699, *Sericopimpla abdominalis* Morl., Tow. 1970, SAM-HYM-P000050 (SAMC). • 1 male, [South Africa, Eastern Cape Province], Burghersdorp [-30.992 26.325], C.P., SA Museum, Mus. Staff, Nov 1939, *Charitopimpla sericata* Kriechb., P.L.G. Benoit det., 1955, SAM-HYM-P000051 (SAMC). • 1 female, [South Africa, Kwazulu-Natal Province], Mfongosi [−28.712, 30.831], Zulul., W.E. Jones, Feb. 1917, SAM-HYM-P000052 (SAMC). • 2 females, ditto except for Mch. 1917, SAM-HYM-P000053 (SAMC). • 1 female, [South Africa, Kwazulu-Natal Province], M’fongosi, Zululand, W.E. Jones, Apr.–Nov. 1934, *Charitopimpla sericata* Kriechb., P.L.G. Benoit det., 1955, SAM-HYM-P000054 (SAMC). • 2 females. Uganda, Kibale National Park, Kanyawara; Tapani Hopkins leg; malaise traps; ZMUT: http://mus.utu.fi/ZMUT.19931, http://mus.utu.fi/ZMUT.13804.

##### Distribution.

Democratic Republic of Congo (Roman 1910; Benoit 1953), Kenya (Townes and Townes 1973), Madagascar (Townes and Townes 1973), Mozambique (Kriechbaumer 1895), Rwanda (Benoit 1955), South Africa (Morley 1916; Benoit 1956), Tanzania (Szépligeti 1908; Townes and Townes 1973), Uganda (new record).

##### Biological notes.

Idiobiont ectoparasitoid of *Kotochalia junodi* (Heylaerts, 1890) larvae (Smithers 1956).

##### Variation.

**Figures 11–14.**
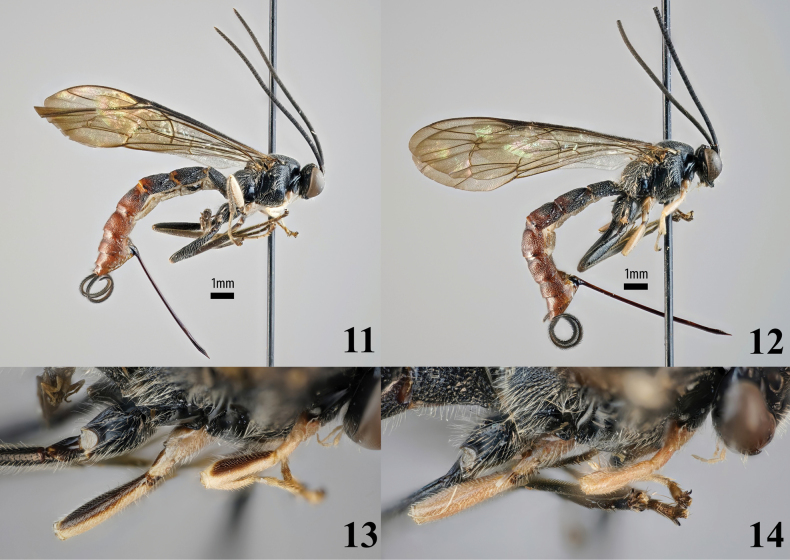
*Sericopimpla sericata*, non-type females (ZMUT). **11, 12**. Habitus, lateral view; **13, 14**. Fore and mid femur, dorsal view; **11, 13**. http://mus.utu.fi/ZMUT.13804; **12, 14**. http://mus.utu.fi/ZMUT.19931.

The two Ugandan specimens have black-marked basal metasomal tergites, similar to *S. kibalensis* sp. nov., whereas the holotype only has the apico-lateral corners of tergite 2–4 black. One Ugandan specimen has, like the holotype, an entirely white tegula and at most weakly brownish postero-lateral fore and mid femurs (Figs 12, 14: http://mus.utu.fi/ZMUT.19931). The other Ugandan specimen has a mostly black tegula and clearly blackish postero-lateral fore and mid femurs (Figs 11, 13: http://mus.utu.fi/ZMUT.13804), resembling the pattern of *S. kibalensis* sp. nov. Overall, the five South African females match the lectotype in colouration, but four of them have some of the tergites darker (in one specimen the apical three tergites are darker, in the other three the darkening is present on the first or second tergite); the single male matches the paratype in colouration.

## Discussion

A study on the ecology and conservation of the pimplines of Kibale National Park, including *Sericopimpla kibalensis* sp. nov., is currently under preparation. Some deductions on the biology of the species are already possible. Based on its being in the same genus as *S. sericata* and being structurally similar (although smaller), *S. kibalensis* sp. nov. presumably attacks bagworm moths or other moth species occupying similar microhabitats. That all 46 specimens of *S. kibalensis* sp. nov. were caught in traps in forest and none in nearby farmland suggests that this species’ local populations need the protection and hosts offered by the forest. *S. kibalensis* sp. nov. seems much more abundant than *S. sericata* in forests in Kibale. However, it may possibly not have as wide a distribution in Africa, since previous sampling (although highly incomplete and likely biased towards non-forest habitats) had not detected the species.

We interpret the differences in the colour of the tegula, fore and mid femur, and basal metasomal tergites of *S. sericata* as intraspecific variation as they are otherwise highly similar and we found no structural differences either between the two Ugandan specimens or between them and photographs of the South African lectotype. If the rather extensively black-marked basal metasomal tergites, for example, are only present in *Sericopimpla* in the Ugandan region, possible specimens of *S. kibalensis* sp. nov. collected elsewhere may turn out to have more orange or yellow basal tergites.

## Supplementary Material

XML Treatment for Sericopimpla kibalensis

XML Treatment for Sericopimpla sericata

## Additional information

### Use of AI

No use of AI was reported.

### Author ORCIDs

Emil M. Österman https://orcid.org/0009-0008-8307-5804

Tapani Hopkins https://orcid.org/0000-0002-2256-0098

Ilari E. Sääksjärvi https://orcid.org/0000-0002-8107-5607

Kari M. Kaunisto https://orcid.org/0000-0001-6665-0047

Simon van Noort https://orcid.org/0000-0001-6930-9741

### Data availability

All images presented in this paper, as well as supplementary images, are available on WaspWeb at https://www.waspweb.org. The data of the Ugandan wasps is in the Zenodo repository (Österman et al. 2025b, https://doi.org/10.5281/zenodo.17855730).
